# Effect of a Se-enriched *Limosilactobacillus fermentum* CGMCC 17434 compound microbial agent synergising with peanut sprouts on the flavor of Se-enriched yogurt

**DOI:** 10.1016/j.fochx.2026.104132

**Published:** 2026-06-23

**Authors:** Zhuhui Tian, Hui Zhou, Xiefei Li, Jie Luo, Yuxing Guo, Zhen Wu, Daodong Pan, Xiankang Fan

**Affiliations:** a School of Public Health, the Key Laboratory of Environmental Pollution Monitoring and Disease Control, Ministry of Education, Guizhou Provincial Engineering Research Center of Ecological Food Innovation, Guizhou Medical University, Guiyang 561113, China; b College of Food Science and Technology, Hunan Agricultural University, Changsha 410114, China; c Key Laboratory of Animal Protein Food Processing Technology of Zhejiang Province, College of Food and Pharmaceutical Sciences, Ningbo University, Ningbo 315832, China; d School of Food Science and Pharamaceutical Engineering, Nanjing Normal University, Nanjing 210023, China

**Keywords:** Lactic acid bacteria, Organic selenium, Plant-based yogurt, Yogurt quality, Flavor compounds

## Abstract

This study investigated the effect of a Se-enriched *Limosilactobacillus fermentum* CGMCC 17434 compound microbial agent synergising with peanut sprouts on the flavor of Se-enriched yogurt. In the synergistic fermentation group, viable counts, titratable acidity, and water-holding capacity reached 8.70 lg CFU/mL, 94.57°T, and 83.82%, respectively. *E*-noses, e-tongues and rheology indicated that, compared with peanut sprout alone, texture and rheological properties improved, umami and richness enhanced, and astringency and bitterness reduced. Additionally, HS-SPME-GC–MS and GC-IMS indicated that adding peanut sprout enriched anethole and 1-hexanol, imparting grassy, fruity and sweet notes. The compound microbial agent promoted pentanoic acid methyl ester and acetic acid ethyl ester and inhibited off-flavor compounds. Metabolomics revealed that differential metabolites were mainly enriched in TCA cycle and amino acid metabolism pathways, with key metabolites including L-glutamic acid, aspartate, L-arginine, and D-proline significantly upregulated. This study provides a theoretical basis for developing Se-enriched yogurt with excellent flavor.

## Highlights

• Compound microbial agent synergising with peanut sprouts improved yogurt flavor.
• Compound microbial agent improved yogurt texture and rheological properties.
• Co-fermentation increased the levels of anethole, 1-hexanol, and methyl valerate.
• TCA cycle intermediates and umami amino acids are the key reason.
• Phospholipid remodeling and antioxidant network enhanced bacterial adaptability.

## Graphical abstract

**Figure:**
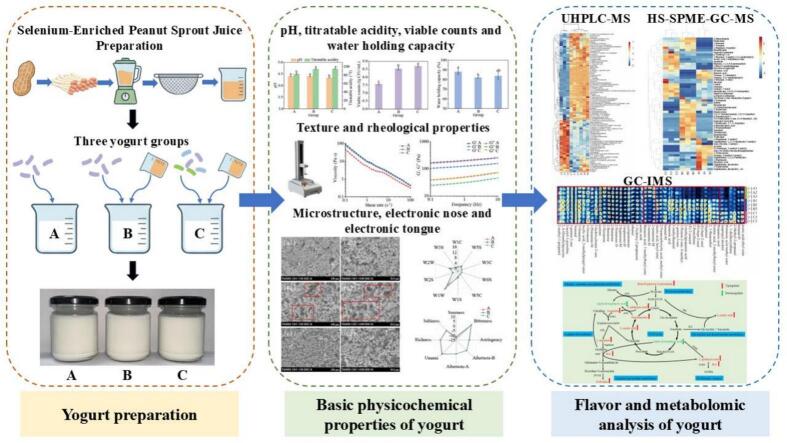

## 1 Introduction

Yogurt, as a widely popular fermented dairy product, is not only rich in nutrients but also possesses multiple health benefits such as regulating intestinal microbiota and enhancing immunity (Routray & Mishra, 2011). Traditional yogurt is typically fermented with *Streptococcus salivarius* subsp. *thermophilus and Lactobacillus delbrueckii* subsp. *Bulgaricus* as starters, and its flavor is dominated by milky and sour notes (Cheng, 2010). However, plain yogurt lacks antioxidants and anti-ageing compounds such as organic selenium, resveratrol and polyphenols, and suffers from issues such as whey separation and a lack of flavor variety (Peng et al., 2022). In recent years, as the ageing population continues to grow, there is an urgent need to develop high-quality yogurt products that offer anti-ageing health benefits and a pleasant taste (Fan et al., 2022).

Selenium is an essential trace element for the human body. As a core component of glutathione peroxidase (GPx), it plays a crucial role in antioxidant defense, immune enhancement, and thyroid function regulation (Bai, Zhang, et al., 2024). However, the human body cannot synthesize selenium by itself, and traditional selenium supplementation methods (e.g., selenium-enriched foods or inorganic selenium supplements) suffer from limitations such as low bioavailability and narrow safety margins (Dinh et al., 2018). Notably, lactic acid bacteria (LAB) possess the ability to convert inorganic selenium into organic selenium. Studies have shown that fermentation with selenium-enriched *Lactiplantibacillus plantarum* NML21 can significantly increase the content of creamy flavor compounds such as 2-heptanone and 2,3-pentanedione in yogurt, enhancing the fruity and milky notes of the product (Wang et al., 2025). Selenium supplementation can also regulate ornithine carbamoyltransferase activity, significantly increasing amino acids such as arginine and leucine, as well as ester flavor compounds, thereby enhancing cheese, banana, and pineapple aromas (Guo et al., 2025). Moreover, in plant-based fermentation systems, LAB have been demonstrated to efficiently degrade common beany aldehydes such as hexanal and nonanal into alcohols or acids via decarboxylation and reduction reactions, effectively reducing earthy, bitter, and astringent off-flavors (Molina et al., 2025). We previously screened traditional fermented foods to identify a highly efficient Se-enriching strain *Limosilactobacillus fermentum* CGMCC 17434, which achieves an organic selenium conversion rate of 88.12% (Wu et al., 2021). When this strain was combined with *Lactiplantibacillus plantarum* CGMCC 1.5953, the resulting yogurt contained 172.10 ± 0.56 μg/L of organic selenium (Luo, Tian, et al., 2025). However, their impact on the quality of selenium-enriched yogurt remains unclear.

Interestingly, during germination, peanuts can convert inorganic selenium into organic selenium, which exhibits higher bioavailability and lower toxicity (Martínez et al., 2020; Pophaly et al., 2014), making them an ideal selenium-enriched plant-based raw material (Zhou et al., 2021). Furthermore, during this process, peanuts can also accumulate resveratrol, phenolic compounds, and various flavor precursors (Luo et al., 2021). Studies have shown that adding plant-based ingredients can significantly alter the flavor profile of yogurt. For example, Yuan et al. (2026) used red bean sprouts combined with probiotics to ferment yogurt, which produced fresh grassy and floral notes and enhanced flavor complexity. Gurkan and Hayaloglu (2017) incorporated basil leaves into yogurt, significantly changing the volatile flavor profile and increasing terpenes such as linalool, but the sensory scores were lower than those of yogurt without basil, indicating that basil leaves may introduce unacceptable flavors while bringing new aromas. Cai et al. (2025) found that mulberry fruit extract together with the probiotic *Lactiplantibacillus plantarum* NUC08 significantly increased flavor-related metabolites in yogurt, enriching the fruity characteristics of the yogurt. These studies indicate that plant-based ingredients provide a broad space for developing specialty flavored yogurts. However, plant materials themselves may also introduce undesirable notes such as green, grassy, and earthy off-flavors. We previously established a method for producing selenium-enriched peanut seedlings and found that fermented milk rich in organic selenium can reverse the D-galactose-induced upregulation of p-ERK/ERK, p-CREB/CREB and BDNF expression in the hippocampus of aged mice, thereby delaying the ageing process and cognitive impairment in these mice (Luo, Zhuang, et al., 2025; Wu et al., 2021). However, the effects of L. *fermentum* CGMCC 17434 compound microbial agent and peanut sprouts on the quality of Se-enriched yogurt, particularly its flavor characteristics, remain unclear. Consequently, we hypothesise that they may exhibit a synergistic effect during the co-fermentation of selenium-enriched yogurt, thereby enhancing the yogurt's flavor.

This study investigated the effect of a Se-enriched *Limosilactobacillus fermentum* CGMCC 17434 compound microbial agent synergising with peanut sprouts on the flavor of Se-enriched yogurt. Firstly, the study investigated their effects on the live bacterial count, pH, titratable acidity (TA), color, water-holding capacity (WHC), texture, rheological properties and microstructure of fermented milk. Secondly, the effects on the flavor of fermented milk were characterised using an electronic nose, an electronic tongue, HS-SPME-GC–MS and GC-IMS. Finally, UHPLC-MS combined with metabolomics analysis was employed to elucidate the molecular mechanisms underlying these effects. The findings provides a theoretical basis for developing Se-enriched yogurt with excellent flavor.

## 2 Materials and methods

### 2.1 Materials

*L. fermentum* CGMCC 17434 and *L. plantarum* CGMCC 1.5953 were screened by Ningbo University and deposited in China General Microbiological Culture Collection Center (CGMCC). The starter (containing *Streptococcus salivarius* subsp. *thermophilus* and *Lactobacillus delbrueckii* subsp. *Bulgaricus*) was produced by Wecare Probiotics (Suzhou, China) Co., Ltd. Peanuts, pure milk, sucrose, and sodium selenite were produced by Shouguang Science and Technology College (Weifang, China), Inner Mongolia Mengniu Dairy Co., Ltd., (Inner Mongolia, China), Shanghai Xinglian Biotechnology Co., Ltd. (Shanghai, China), and Shanghai Yuanye Biotechnology Co., Ltd. (Shanghai, China), respectively.

### 2.2 Yogurt preparation

Selenium-enriched peanut sprout juice was prepared as described by Luo, Tian, et al. (2025). Briefly, peanuts were soaked in 12 mg/L sodium selenite solution at 30 °C for 10 h and germinated at 28 °C until sprouts reached 3 cm, then washed, blanched for 5 min, cooled, homogenised with milk (1:2, *w*/*v*), and filtered through a 180-mesh sieve. The first starter was prepared by adding 1.0% (*w*/*v*) starter to sterilized milk and fermenting at 37 °C for 6 h. The second and third starters were prepared by inoculating L. *fermentum* CGMCC 17434 and L. *plantarum* CGMCC 1.5953 into sterilized milk at 3.0% (*v*/v), respectively, followed by fermentation at 37 °C for 12 h. All starters were subcultured 3–4 times before use. The three yogurt groups were prepared as follows: Group A contained 8.0% (w/v) sucrose and 6% (v/v) first starter; Group B additionally contained 40% (v/v) peanut sprout juice; Group C additionally contained 40% (v/v) peanut sprout juice and 6% (v/v) compound microbial agent (the first, second, and third starter at a ratio of 2:3:1). All groups were fermented at 42 °C for 4–6 h and then refrigerated at 4 °C for 24 h for post-ripening.

### 2.3 Effect on viable counts, pH and titratable acidity

Following the method described by Peng et al. (2022), the viable counts of various LAB strains in yogurt were determined using selective media combined with specific culture conditions. Yogurt samples were serially diluted 10-fold with a 0.9% NaCl solution, and three consecutive appropriate dilutions were selected for counting using the plate spread method. The pH value of the yogurt was measured using a pH meter (PHS-3C, China). The determination of TA was performed according to the method described by Tian et al. (2017), with modifications. A 10 g of yogurt was weighed, mixed with 20 mL of sterile water and 0.2 mL of 1% phenolphthalein indicator, and then titrated with 0.1 mol/L NaOH standard solution to the endpoint. TA was calculated using the following equation:

(1) TA%=V×C×0.09×100m#

Where, V is the volume of NaOH solution consumed in the titration (mL), C is the molar concentration of NaOH (mol/L), m is the mass of the yogurt sample (g), and 0.09 is the millimolar mass of lactic acid (90 g/mol ÷ 1000).

### 2.4 Effect on color and water holding capacity

The L*, a*, and b* values of yogurt were measured using a colorimeter (CS-580 A, CHN Spec, China), and the instrument was calibrated before measurement. The WHC was determined according to the method of Wu et al. (2023) with slight modifications. A 10 *g* yogurt was centrifuged at 4 °C and 5000 rpm for 10 min, and the supernatant was discarded. The WHC was calculated using the following formula:

(2) $$\begin{array}{c} \mathrm{WHC}\;\left(\%\right)=\frac{m2}{m1}\times 100\# \end{array}$$

Where, m_1_ represents the initial weight of the yogurt, and m_2_ represents the weight of the yogurt after centrifugation and removal of the supernatant.

### 2.5 Effect on texture and rheological properties

The texture of yogurt was determined according to the method described by Fan, Liu, et al. (2024) using a texture analyzer (Stable Micro Systems, UK). The detection parameters were as follows: probe pause time 2 s, trigger force 5 g, strain 50%, the probe was raised to a height of 80 mm above the sample surface, pre-test speed 2 mm/s, test speed 1 mm/s, post-test speed 2 mm/s. The hardness, adhesion, cohesion, elasticity, gumminess and chewiness of yogurt were determined. The rheological properties of yogurt were measured using a rotational rheometer (Kinexus pro+, Malvern, UK) according to the method described by Benchabane et al. (2024). The temperature was 25 °C, the shear rate ranged from 0.01 to 100 s^−1^, the fixed frequency was 1 Hz, the strain was 1%, and the frequency ranged from 0.1 to 100 Hz.

### 2.6 Effect on microstructure

The microstructure of yogurt was examined according to the method described by Nguyen et al. (2018) with modifications. A 1 cm × 1 cm × 1 cm yogurt sample was taken and fixed overnight in 2.5% (*v*/v) glutaraldehyde at 4 °C. Following fixation, it was dehydrated sequentially with 50%, 60%, 70%, 80%, and 90% ethanol solutions for 10 min each, and then eluted three times with 100% ethanol for 15 min each. The dehydrated samples were immersed in tert-butanol three times for 15 min each, pre-cooled at −80 °C for 24 h, and then freeze-dried. After drying, the samples were retrieved, gold-coated, and examined for surface morphology using a TM4000 II scanning electron microscope (Hitachi High-Tech, Japan).

### 2.7 Effects on electronic tongue and electronic nose

Electronic tongue determination was performed according to the modified method of Han et al. (2025). Yogurt was diluted with an equal volume of deionized water, transferred to a 50 mL centrifuge tube, and centrifuged at 6000 rpm for 10 min. The supernatant was collected and analyzed using an electronic tongue (SA402B Plus-EX, INSENT, Japan). Electronic nose determination was carried out following the adjusted procedure of Tian et al. (2020). A 20 g aliquot of yogurt was weighed into a centrifuge tube and sealed with plastic wrap. After equilibration at room temperature for 30 min, the headspace gas was extracted and analyzed using an electronic nose (PEN3, AIRSENSE, Germany).

### 2.8 Determination of volatile flavor compounds based on HS-SPME-GC–MS

According to the method of Bao et al. (2025) with some modifications, volatile flavor compounds were extracted from 5 g of yogurt sample in a headspace vial. Extraction was performed at 55 °C and 350 ×g for 1 h. The extracted compounds were analyzed using an Agilent 8890 gas chromatograph coupled with a 5977B mass spectrometer (Agilent, Santa Clara, CA, USA). Helium was used as the carrier gas at a flow rate of 1.0 mL/min, and the injector temperature was maintained at 250 °C. The column temperature was initially set at 35 °C and held for 5 min, then increased to 140 °C at 5 °C/min and held for 2 min, and finally raised to 250 °C at 10 °C/min and held for 3 min.

### 2.9 Construction of flavor fingerprints based on GC-IMS

Gas Chromatography-Ion Mobility Spectrometry (GC-IMS, FlavourSpec® Flavor Analyzer, Anyut, Berlin, Germany) was employed as described by Wang, Fan, et al. (2023). A 5.0 mL yogurt sample was placed in a 20 mL headspace vial, incubated at 80 °C for 20 min, and then 500 μL of the headspace was injected. The analysis time was 30 min. The column type was MXT-5, 15 m long with an inner diameter of 0.53 mm and a film thickness of 1 μm. Column temperature was 40 °C, carrier gas/drift gas was N₂, and IMS temperature was 45 °C. Incubation time was 20 min at 80 °C, with an injection needle temperature of 85 °C and incubation speed of 500 rpm.

### 2.10 Determination of nonvolatile flavor compounds based on UHPLC-MS

Following the method described by Gu et al. (2020) with minor modifications, 1 mL of yogurt was transferred into a 2 mL centrifuge tube, mixed with 400 μL of methanol, and vortexed for 1 min. The mixture was centrifuged at 12,000 ×*g* for 10 min at 4 °C. The supernatant was collected and concentrated to dryness. The residue was re-dissolved in 150 μL of 2-chloro-*L*-phenylalanine solution (4 ppm) prepared in 80% methanol-water. After filtration through a 0.22 μm membrane, the sample was subjected to Ultra-High-Performance Liquid Chromatography-Mass Spectrometry (UHPLC-MS) analysis. The system was equipped with a Thermo Vanquish UHPLC system (Thermo Fisher Scientific, Waltham, MA, USA) coupled with an ACQUITY UPLC® HSS T3 column (2.1 × 100 mm, 1.8 μm, Waters, Milford, MA, USA). The flow rate was set at 0.3 mL/min, column temperature at 40 °C, and injection volume at 2 μL. Data were acquired using a Thermo Q Exactive Focus mass spectrometer (Thermo Fisher Scientific, USA). The spray voltage was set to 3.50 kV in positive ion mode and − 2.50 kV in negative ion mode.

### 2.11 Statistical analysis

All experiments were conducted independently at least three times. SPSS software was used for one-way analysis of variance and Duncan multiple range test to analyze the differences between samples. Origin software, Simca software and BioLadder (https://www.bioladder.cn), Omicstudio (https://www.omicstudio.cn), (https://cloud.metware.cn) were used for data visualization.

## 3 Results and discussion

### 3.1 Effect on viable counts, pH and titratable acidity

**Fig. 1:**
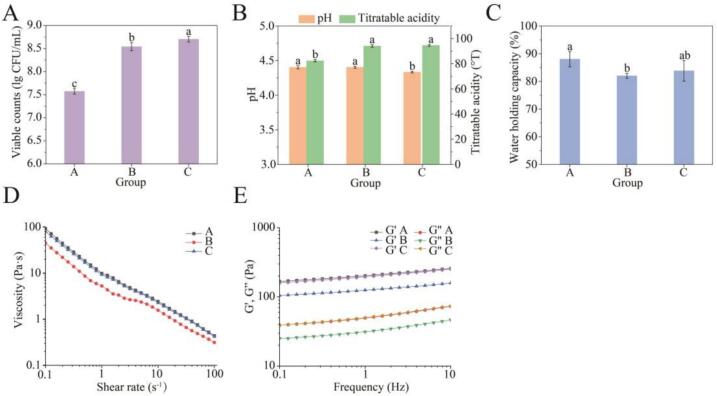
Effects on the Physical and Chemical Properties of Yogurt. (A) Viable counts. (B) pH and titratable acidity. (C) Water holding capacity. (D) Apparent viscosity. (E) Frequency scanning. Different letters “a, b, c” indicate significant differences between groups (*P* < 0.05).

The addition of selenium-enriched peanut sprout juice and compound microbial agent significantly affected the viable counts, pH, and TA of the yogurt. As shown in Fig. 1-A, the viable counts in groups B and C reached 8.54 ± 0.09 and 8.70 ± 0.06 lg CFU/mL, respectively, which was significantly higher than group A(*P* < 0.05). The higher viable counts in groups B and C can be attributed to the abundant nutrients, such as amino acids, vitamins, and minerals, present in the peanut sprout juice, which promoted the growth of LAB (Song et al., 2023; Wang et al., 2026; Yu et al., 2022). Furthermore, studies have shown that selenium can enhance the growth and viability of LAB (Zan et al., 2024), and El-Sayed et al. (2022) found that the incorporation of selenium-enriched ingredients increased probiotic counts in yogurt. Notably, the viable counts in group C were significantly higher than that in group B, indicating a synergistic effect of the compound microbial agent. This synergism may have facilitated more efficient utilization of the nutrients in the peanut sprout juice, thereby further promoting bacterial proliferation (Fan, Zhang, et al., 2024).

The pH measurement results (Fig. 1-B) showed that the pH value of group C was significantly lower than those of groups A and B (*P* < 0.05), on the contrary, the TA was the highest. The decrease in pH and the increase in TA directly reflect the extent of LAB metabolism and acid production. The lower pH values in groups B and C are attributed to the abundant nutrients provided by the peanut sprout juice, which effectively promoted the metabolic activity of the LAB. The pH value in group C was the lowest, which is consistent with its highest viable counts. A higher number of viable bacteria implies stronger metabolic capacity and acid production rate, consequently resulting in a lower final pH.

### 3.2 Effect on color and water holding capacity

The color of the three yogurt groups is presented in Table 1. The L* (black-white) value of group A was significantly higher than those of groups B and C, which were supplemented with selenium-enriched peanut sprout juice (*P* < 0.05). This reduction in brightness is attributed to the presence of colored compounds, such as flavonoids and phenolics in the peanut sprout juice (Zhou et al., 2021). Regarding the a* (red-green) and b* (yellow-blue) values, group A exhibited the typical milky-white color of yogurt with a slight greenish tinge (negative a* value) and a faint yellow hue (positive b* value) (Daszkiewicz et al., 2024). Groups B and C showed significantly lower a* values and significantly higher b* values (*P* < 0.05), imparting a deeper yellowish-green tone to the yogurt. Notably, the a* value of group B was significantly lower than that of group C (*P* < 0.05), indicating that group B exhibited a greener color than group C. This difference may be explained by two factors. First, the lower pH environment in group C may have induced slight degradation or transformation of the green pigments present in the peanut sprout juice (Koca et al., 2007), thereby attenuating the green tone. Second, glycosidases such as β-glucosidase produced by LAB have been shown to convert anthocyanin pigments into phenolic acid metabolites (Braga et al., 2018; Cheng et al., 2016).

**Table 1:** The color of yogurt. Different letters “a, b, c” indicate significant differences between groups (*P* < 0.05).

Group	L^⁎^	a^⁎^	b^⁎^
A	84.123 ± 0.789^a^	−0.173 ± 0.095^a^	2.740 ± 0.108^b^
B	82.810 ± 0.120^b^	−0.627 ± 0.035^c^	8.107 ± 0.050^a^
C	82.713 ± 0.032^b^	−0.393 ± 0.031^b^	8.043 ± 0.196^a^

WHC is a key indicator for evaluating the stability of the yogurt gel network and its ability to prevent whey separation (Godoi et al., 2021). As shown in Fig. 1-C, group A exhibited the highest WHC, which was significantly higher than that of group B (*P* < 0.05), but showed no significant difference compared with group C (*P* > 0.05). The reduction in WHC may be attributed to the dilution effect of the added peanut sprout juice on the protein gel network, which decreased the relative concentration of casein (Li et al., 2025; Oliveira et al., 2022). In addition, phenolic and flavonoid compounds introduced by the peanut sprout juice may interfere with intermolecular protein cross-linking (Zhang et al., 2025), hindering normal gel network aggregation and leading to a looser structure with reduced water entrapment capacity (Wang, Fan, et al., 2023; Zou et al., 2025). However, despite the addition of the same amount of peanut sprout juice, the WHC of group C recovered to a level comparable to that of group A, indicating that compound microbial agent played a key role in this improvement. Abundant small molecules (organic acids, amino acids, and small peptides) were generated by compound microbial agent metabolism. These molecules modulated intermolecular interactions (hydrophobic, hydrogen bonding, and electrostatic) between proteins (Bai, Yang, et al., 2024; Yang et al., 2024), thereby promoting a more compact and uniform gel network.

### 3.3 Effect on texture and rheological properties

Texture properties are core indicators for evaluating the mouthfeel and sensory quality of yogurt, and the different treatments significantly influenced these parameters. As revealed in Table 2, hardness, gumminess, and chewiness were significantly higher in group A than in groups B and C (*P* < 0.05), with no significant differences observed between groups B and C (*P* > 0.05). This indicates that the addition of peanut sprout juice diluted the relative protein concentration, which was the primary factor responsible for the decreased gel strength. This finding is consistent with Zhou et al. (2025), who reported similar effects of ginseng addition on yogurt protein content and hardness. Cohesiveness was highest in group C, while adhesiveness and resilience followed the order B > C > A (*P* < 0.05). This may be attributed to the exopolysaccharides (EPS) produced by the compound microbial agent, which hindered syneresis, improved the structural integrity of the gel network, and enhanced the viscosity of the system (Brüls et al., 2024; Ramos et al., 2023). Numerous LAB strains, such as *Lactiplantibacillus plantarum* and *Lacticaseibacillus casei*, have been confirmed to produce EPS (Genc et al., 2024), and EPS can significantly increase the adhesiveness and hardness of yogurt through interactions with proteins (Wu et al., 2025). Overall, these findings suggest that compound microbial agent effectively mitigated the negative impact of peanut sprout juice on texture properties.

**Table 2:** The texture of yogurt. Different letters “a, b, c” indicate significant differences between groups (*P* < 0.05).

Group	Hardness	Adhesiveness	Springiness	Cohesiveness	Gumminess	Chewiness	Resilience
A	135.959 ± 15.396^a^	−543.233 ± 53.136^c^	0.969 ± 0.002^a^	0.424 ± 0.014^c^	57.685 ± 6.802^a^	55.904 ± 6.589^a^	0.020 ± 0.001^c^
B	95.162 ± 11.737^b^	−292.356 ± 45.964^a^	0.963 ± 0.001^b^	0.445 ± 0.005^ab^	42.317 ± 5.154^b^	40.731 ± 4.928^b^	0.027 ± 0.002^a^
C	99.045 ± 8.393^b^	−395.523 ± 54.695^b^	0.960 ± 0.004^b^	0.461 ± 0.012^a^	45.725 ± 4.982^b^	43.887 ± 4.970^b^	0.023 ± 0.001^b^

As shown in Fig. 1-D, the viscosity of the three yogurt groups decreased with increasing shear rate, exhibiting typical shear-thinning behavior. This characteristic contributes to the good swallowability and mouthfeel of yogurt (Atik et al., 2024). Over the entire shear rate range, the apparent viscosity followed the order A > C > B, with the viscosity curve of group C lying above that of group B and close to that of group A. Frequency sweep results (Fig. 1-E) showed that across the entire tested frequency range, the storage modulus (G') was higher than the loss modulus (G") for all three groups, and G' and G" values followed the same order A > C > B. Hou et al. (2024) reported that in mixed polysaccharide-protein systems, the formation of an interpenetrating polymer network can significantly enhance gel homogeneity and network density, thereby improving rheological properties and WHC. This improvement in group C can be attributed to EPS, which were produced by compound microbial agent metabolism and filled the interstitial spaces within the protein network, enhancing water hydration capacity. Consequently, gel strength and viscosity increased, a finding that is consistent with the texture analysis results.

### 3.4 Effects on microstructure

**Fig. 2:**
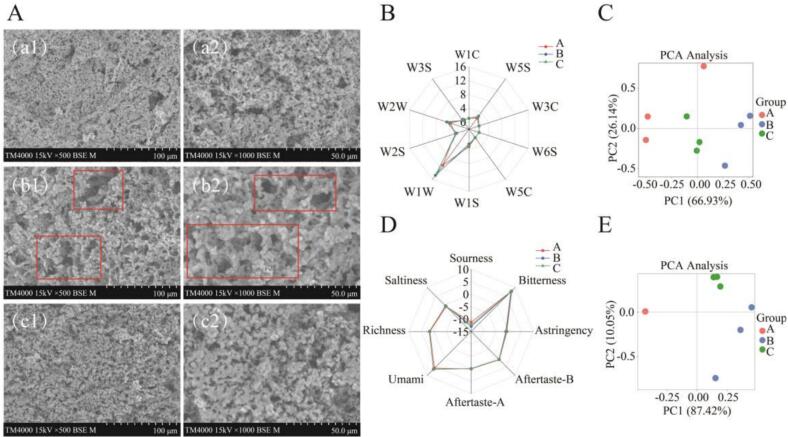
Microstructural and sensory characterization of yogurt under different treatments. (A) Scanning electron microscopy (SEM) images were captured at magnifications of ×500 and ×1000 (15 kV). Panels a1 and a2 correspond to Group A, panels b1 and b2 to Group B, and panels c1 and c2 to Group C. (B) Electronic nose radar plot. (C) PCA score plot of electronic nose data. (D) Electronic tongue radar plot. (E) PCA score plot of electronic tongue data.

The SEM images (Fig. 2-A) intuitively illustrate the impact of different treatments on the microstructure of yogurt. Group A exhibited the typical microstructure of a yogurt gel, where casein micelles aggregated to form a dense, continuous three-dimensional network with uniform pore distribution and small pore sizes (Heiden-Hecht et al., 2023). In contrast, the addition of peanut sprout juice in group B resulted in a noticeably looser gel network with larger and unevenly distributed pores. Localized areas showed signs of network fracture or collapse, indicating a decline in overall structural integrity. This sparse network scaffold was unable to effectively retain water, and its resistance to deformation was consequently diminished. These observations directly explain the reduced hardness, storage modulus, and WHC observed in group B. In contrast, group C largely maintained a uniform and dense network structure. Combined with the earlier texture and rheological results, it can be inferred that the introduction of compound microbial agent may have repaired the structural loosening caused by peanut sprout juice. This repair was achieved by modulating protein cross-linking, polysaccharide distribution, and the accumulation of metabolic products.

### 3.5 Effects on electronic tongue and electronic nose

The electronic nose measurement results (Fig. 2-B) reflected the differences in volatile flavor components among the different yogurt treatment groups. PCA analysis (Fig. 2-C) showed that PC1 and PC2 accounted for 66.93% and 26.14% of the variance, respectively, with a cumulative explanation rate of 93.07%, indicating that the addition of peanut sprout juice and probiotics significantly altered the overall flavor profile of the yogurt. The response values for W1C (aromatics), W1S (methyl groups), and W2S (alcohols) all followed the order A > C > B. Specifically, group A showed responses that were 5.54%, 25.23%, and 18.02% higher than group B, and 2.12%, 13.04%, and 5.32% higher than group C, respectively. This suggests that group A was richer in aromatic, alcohol, and alkane compounds. The addition of peanut sprout juice diluted these flavor components, while the smaller differences between groups C and A may be related to the metabolic regulation by compound microbial agent. In contrast, the response values for W5S (nitrogen oxides), W1W (sulfides), and W2W (aromatics and organosulfides) followed the order B > C > A. The plant-derived characteristic flavor introduced by peanut sprout juice added a unique flavor dimension to the yogurt, attributed to stilbenes, terpenes, and nitrogen-containing metabolites, which are mainly associated with grassy, beany, and woody notes. However, when these compounds accumulate in excess, they can cause undesirable off-flavors and disrupt the aroma balance, thereby reducing overall sensory acceptability (Zhu et al., 2024). Compared with group B, the response values in group C decreased by 6.71%, 4.78%, and 9.46%, respectively, indicating that while retaining the plant-derived flavors, the compound microbial agent partially metabolized and transformed them, resulting in a more harmonious and balanced overall flavor profile.

The electronic tongue analysis results are shown in Fig. 2-D, and principal component analysis (Fig. 2-E) revealed that PC1 contributed 87.42% and PC2 contributed 10.05%, with a cumulative explanation rate of 97.47%. Sourness, bitterness, and bitter aftertaste all followed the order A > C > B. The sourness trend differed somewhat from the measured pH and TA, possibly due to the differential sensitivity of the electronic tongue's sourness sensors to various organic acid types and buffer systems (Raj et al., 2025). The reduction in bitterness and bitter aftertaste may be attributed to the interaction between polyphenols in the peanut sprout juice and proteins, which reduces the exposure of bitter peptides, as well as to the degradation of bitter peptides by LAB (Shimamura et al., 2009). Astringency and astringent aftertaste were both higher in group A than in groups B and C, likely because proteins in the peanut sprout juice formed complexes with polyphenolic compounds, thereby reducing astringency perception (Lai et al., 2025). In terms of umami and richness, both groups B and C were higher than group A, with group C achieving the highest richness. This can be mainly attributed to the free amino acids abundant in the peanut sprout juice, as well as the umami peptides and flavor nucleotides produced during fermentation. The co-metabolism of the compound microbial agent further promoted protein hydrolysis and amino acid metabolism, thereby enhancing umami and richness (Spaccasassi et al., 2024). Saltiness followed the order B > C > A, which may be due to mineral ions (such as sodium, potassium, and magnesium) introduced by the selenium-enriched peanut sprout juice. The slightly lower saltiness in group C compared to group B may be associated with the metabolic transformation of selenium by the compound microbial agent, where some inorganic selenium was converted into organic selenium forms, thereby reducing the relative concentration of free sodium ions or altering the existing forms of the mineral ions (Yuan et al., 2025).

### 3.6 Volatile flavor compounds

**Fig. 3:**
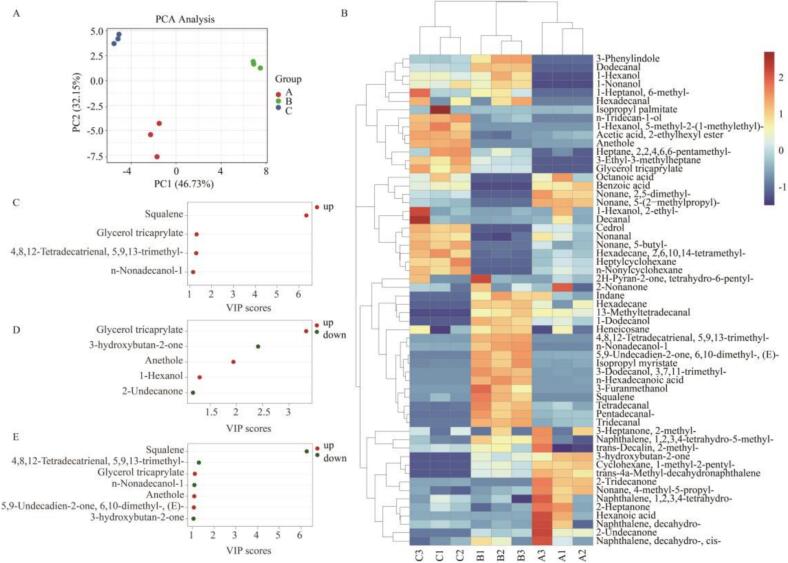
Analysis of volatile flavor compounds in yogurt by HS-SPME-GC–MS. (A) PCA score plot. (B) Heatmap of volatile flavor compounds. (C) VIP scores of differential compounds between group A and B. (D) VIP scores of differential compounds between group A and C. (E) VIP scores of differential compounds between group B and C.

Volatile flavor compounds in the yogurt samples were detected using GC–MS combined with headspace solid-phase microextraction (HS-SPME). As shown in Fig. 3-A, PCA results revealed that PC1 contributed 46.2% and PC2 contributed 32.23%, with a cumulative contribution rate of 78.43%. The odor activity values (OAVs) of the volatile compounds were calculated to evaluate their potential sensory contributions (Supplementary Table S1). Differential volatile flavor compounds were identified using the screening criteria of VIP ≥ 1, *P* < 0.05, and FC ≥ 1.5 or FC ≤ 0.667. As illustrated in Fig. 3-B, a total of 63 volatile flavor compounds were identified across the three groups, including alcohols, aldehydes, ketones, acids, and esters.

As shown in Fig. 3-C, four compounds were significantly upregulated in group B compared with group A: squalene, glycerol tricaprylate, 4,8,12-tetradecatrienal, 5,9,13-trimethyl-, and n-Nonadecanol-1. Squalene, a triterpene widely present in vegetable oils and legumes, exhibits antioxidant and anti-tumor activities (Ryan et al., 2007; Yao et al., 2024). Its odor is relatively faint, contributing a mild fatty note. Glycerol tricaprylate is a medium-chain triglyceride naturally occurring in plant oils and imparts a fatty aroma. Intestinal morphology improvement, digestive enzyme activity enhancement, short-chain fatty acid concentration increase, and intestinal barrier-related protein expression upregulation were reported to be induced by this compound, while inflammatory factors and harmful gut microbiota abundance were reduced (Li et al., 2023). 4,8,12-Tetradecatrienal, 5,9,13-trimethyl- is a typical plant-derived terpenoid aldehyde that exhibits grassy, fatty, waxy and slightly woody notes (Rohloff & Bones, 2005). It has the ability to inhibit fungal growth and leads to the accumulation of squalene epoxide (Goldman et al., 1996). N-Nonadecanol-1 is a long-chain fatty alcohol naturally present in plant essential oils and is an important aroma compound of *Neotinea ustulate* (D'auria et al., 2022). The addition of peanut sprout juice introduces characteristic plant-derived volatile flavor compounds into the yogurt, thereby imparting grassy, woody, fatty and floral notes.

As shown in Fig. 3-D, compared with group A, five differential volatile flavor compounds were identified in group C, among which glycerol tricaprylate, anethole, and 1-hexanol were significantly upregulated, while 3-hydroxybutan-2-one and 2-undecanone were significantly downregulated. The upregulation of glycerol tricaprylate, anethole (sweet, anise, licorice) and 1-hexanol (green, fruity, apple-skin and oily) indicates the successful introduction of plant-derived flavor notes from peanut sprouts. 3-Hydroxybutan-2-one, a key creamy flavor compound in yogurt, is a metabolite produced by LAB using glucose as a carbon source. Xu et al. (2025) found in *Lactococcus lactis* N8 that 3-hydroxybutan-2-one can be reutilized as a carbon reserve. Therefore, the decrease observed in group C may be partly explained by its consumption as an alternative carbon source when LAB entered a carbon-limited phase after vigorous metabolism. 2-Undecanone (waxy, fruity, with creamy cheese-like notes) has been reported to produce oxidative off-flavors during milk storage (Xi et al., 2023), and its downregulation in group C makes the main flavor profile purer and more prominent. Ultimately, group C successfully retains the sweet plant aroma from peanut sprout juice while effectively reducing undesirable notes, achieving an overall harmonious and optimized flavor.

As shown in Fig. 3-E, comparing group C with group B, a total of seven differential volatile flavor compounds were identified. Among them, (E)-6,10-dimethyl-5,9-undecadien-2-one (floral, fruity, tropical, green, pear, apple, banana, citrus), glycerol tricaprylate, and anethole were significantly upregulated, while squalene, 4,8,12-tetradecatrienal-5,9,13-trimethyl-, n-nonadecanol-1, and 3-hydroxybutan-2-one were significantly downregulated. Plant-derived volatile compounds tend to impart green, fatty, and raw notes when present at high levels, their significant downregulation reflects the efficient modification and metabolic transformation by the compound microbial agent. In summary, the introduction of peanut sprout juice brought sweet, licorice, floral and fruity notes to the yogurt in group C, while the vigorous metabolism of the compound microbial agent maintained the plant-derived volatiles at an appropriate level. Excessive green and fatty off-flavors were thereby avoided, while pleasant aroma components were highlighted, achieving a high degree of flavor balance and harmony.

### 3.7 Construction of flavor fingerprints

**Fig. 4:**
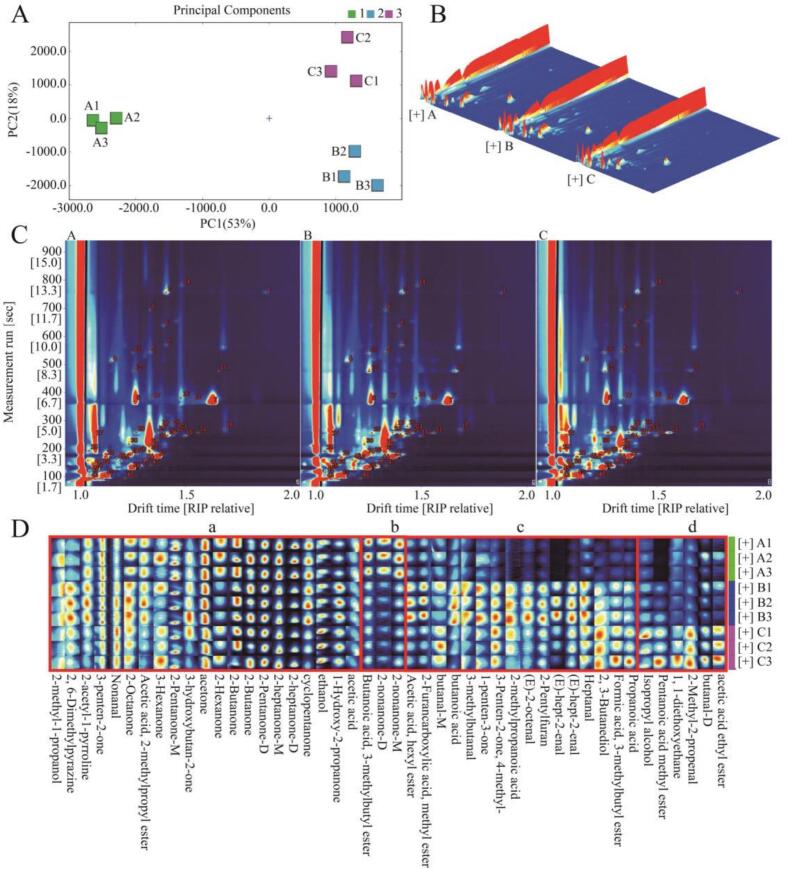
Volatile flavor profiling of yogurt by GC-IMS. (A) PCA score plot. (B) 3D topographic maps. (C) 2D topographic top-view plots. (D) Flavor fingerprinting.

The volatile flavor components of the three yogurt groups were analyzed using GC-IMS. The PCA analysis (Fig. 4-A) showed that PC1 contributed 53% and PC2 contributed 18%. The 3D plot (Fig. 4-B) and top view (Fig. 4-C) further illustrated the differences in volatile flavor profiles among the different treatment groups. The results demonstrated that the three sample groups exhibited distinguishable characteristic peak patterns in the GC-IMS spectra, confirming that the addition of peanut sprout juice and the application of probiotics significantly altered the volatile flavor composition of the yogurt.

According to the flavor fingerprint constructed by GC-IMS (Fig. 4-D), a total of 46 volatile compounds were identified, mainly including aldehydes, alcohols, ketones, acids, and esters. Based on signal intensity, these compounds could be divided into four characteristic regions. Region “a” comprises volatile compounds common to all three groups, including 2-heptanone (cheese, fruity, coconut), 3-hydroxybutan-2-one (creamy, milky, sweet, buttery), acetic acid (sour, fruity), 2-butanone (slightly fruity, green), 2-pentanone (sweet, fruity, banana-like with a fermented nuance), 2-octanone (milky, waxy, cheese), nonanal (aldehydic citrus, cucumber, watermelon rind), 2-acetyl-1-pyrroline (popcorn, toasted grain, malty), and 2,6-dimethylpyrazine (nutty, coffee, bread). These compounds constitute the basic flavor skeleton of yogurt, collectively imparting typical milky, fruity, and roasted notes. Region “b” shows the highest signal intensity in group A, mainly including 2-nonanone (cheesy, green, fruity) and butanoic acid, 3-methylbutyl ester (waxy, fruity). Region “c” contains compounds with significantly higher contents in groups B and C than in group A, including 2-furancarboxylic acid methyl ester (sweet caramel, brown sugar), acetic acid hexyl ester (fruity, green, fresh), propanoic acid (acidic, milky with a pronounced fruity lift), 2,3-butanediol (fruity, creamy, buttery), heptanal (green, grassy, peel), (*E*)-hept-2-enal (green, sweet, fresh fruity), (E)-2-octenal (sweet, green, citrus peel), 2-methylpropanoic acid (acidic, cheesy, creamy), 4-methyl-3-penten-2-one (nutty, earthy), 3-methylbutanal (fruity, dry green, nutty), and butanoic acid (acidic, milky with fruity nuance). These compounds endow the yogurt with complex fruity, grassy, sweet, and fresh notes. Region “d” exhibits the highest signal intensity in group C, including acetic acid ethyl ester (sweet, with grape and cherry nuances), 1,1-diethoxyethane (nutty), and pentanoic acid methyl ester (sweet, ripe fruity, milky). This indicates that the compound microbial agent promoted ester synthesis (Nicolotti et al., 2025), significantly enhancing the fruity character. Meanwhile, cyclopentanone, which contributes to pungent minty notes, was decreased in Group C (Molina et al., 2025).

### 3.8 Nonvolatile flavor compounds

**Fig. 5:**
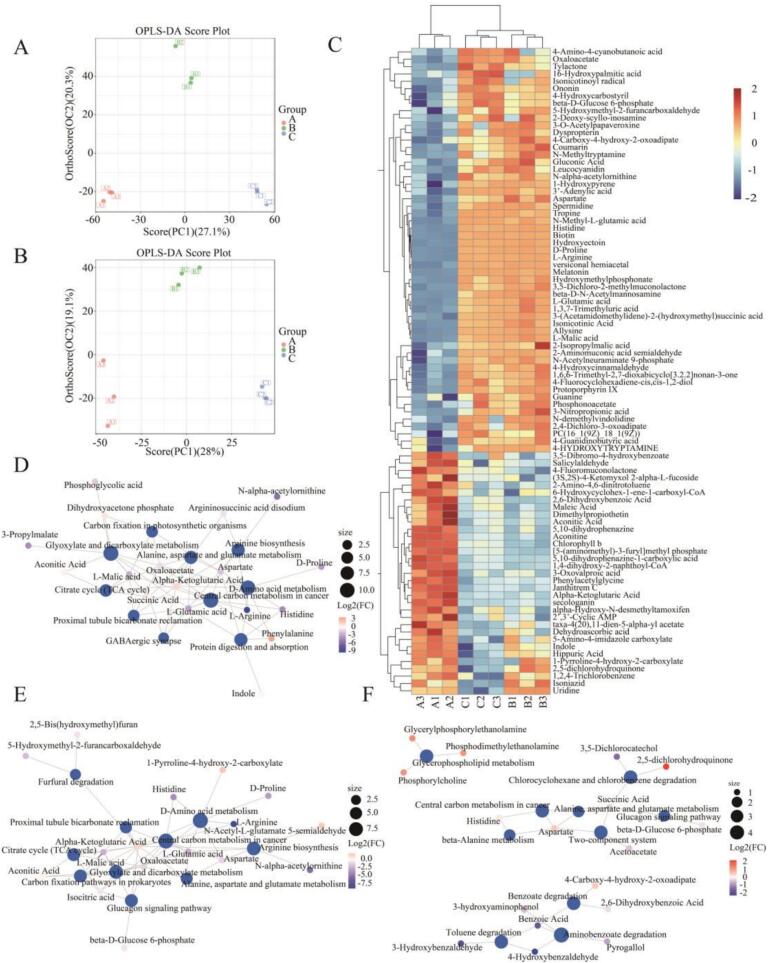
Metabolomic analysis of yogurt by UHPLC-MS. (A) OPLS-DA score plot (positive ion mode). (B) OPLS-DA score plot (negative ion mode). (C) Heatmap of differential metabolites. (D) Metabolic pathway map between Group A and Group B. (E) Metabolic pathway map between Group A and Group C. (F) Metabolic pathway map between Group B and Group C.

Untargeted metabolomics analysis based on UHPLC-MS, combined with OPLS-DA analysis, revealed that the score plots under both positive and negative ion modes (Fig. 5-A, B) showed clear separation of the three sample groups in the score space, with high aggregation of sample points within each group. These results indicate that the addition of peanut sprout juice and the application of probiotics significantly altered the metabolite profile of the yogurt, with pronounced differences observed among the different treatments, as illustrated in the heatmap (Fig. 5-C). Differential metabolites were screened using the criteria of VIP ≥ 1, *P* < 0.05, and FC ≥ 1.5 or FC ≤ 0.667.

Comparing group B with group A, a total of 41 differential metabolites were identified, including 20 significantly upregulated and 21 significantly downregulated metabolites. Among the upregulated metabolites, hydroxyectoine, L-arginine, beta-D-*N*-acetylmannosamine, 3-(acetamidomethylidene)-2-(hydroxymethyl) succinic acid, protoporphyrin IX, spermidine, melatonin, N-alpha-acetylornithine, and histidine were increased by 1398.42, 665.84, 655.74, 180.10, 294.73, 62.57, 50.10, 44.31, and 18.80-fold, respectively. Hydroxyectoine functions as an osmoprotectant, helping to maintain cellular integrity under stress conditions. The upregulation of L-arginine and its derivative N-alpha-acetylornithine points to the activation of the arginine deiminase pathway, which generates ATP and enhances acid tolerance (Teixeira et al., 2014; Yang et al., 2025), while also contributing to the production of flavor-related compounds such as citrulline and ornithine. Previous studies have shown that selenium influences amino acid metabolism in LAB, increasing ornithine carbamoyltransferase activity and elevating levels of amino acids such as citrulline and arginine (Guo et al., 2025), suggesting a potential link between selenium and the activation of arginine metabolism in group B. The accumulation of spermidine and histidine reflects activation of the polyamine synthesis pathway, polyamines possess antioxidant and cytoprotective properties that help LAB resist acid stress during fermentation (Banerji et al., 2022; Yohannes et al., 2005). The upregulation of melatonin and protoporphyrin IX indicates enhanced antioxidant potential of the system (Utaida et al., 2025). Among the downregulated metabolites, phenylalanine, [5-(aminomethyl)-3-furyl] methyl phosphate, pyridoxamine, and alpha-ketoglutaric acid were reduced to 0.08-, 0.13-, 0.26-, and 0.40-fold, respectively. The reduction in alpha-ketoglutaric acid may indicate the consumption of TCA cycle intermediates for amino acid synthesis or energy metabolism (Guan & Liu, 2020). These differential metabolites were primarily enriched in pathways including the TCA cycle, arginine biosynthesis, alanine, aspartate and glutamate metabolism, D-amino acid metabolism, ABC transporters, and furfural degradation (Fig. 5-D).

Compared with group A, a total of 70 differential metabolites were identified in group C, of which 51 were significantly upregulated and 19 were significantly downregulated. Among the upregulated metabolites, hydroxyectoine, L-arginine, L-glutamic acid, L-malic acid, oxaloacetate, 3′-adenylic acid, spermidine, histidine, DL-ornithine, aspartate, and beta-d-glucose 6-phosphate were increased by 1176.49-, 602.75-, 6.66-, 5.25-, 2.37-, 114.34-, 55.31-, 16.54-, 21.52-, 9.86-, and 1.54-fold, respectively. As a direct precursor for gamma-aminobutyric acid (GABA) synthesis (Dhakal et al., 2012), the substantial accumulation of L-glutamic acid provides a metabolic basis for the enhanced umami and richness detected by the electronic tongue in group C. The upregulation of TCA cycle intermediates such as L-malic acid and oxaloacetate indicates an enhanced carbon metabolic flux, supplying sufficient energy and precursors for bacterial proliferation and organic acid accumulation. From an antioxidant perspective, the upregulation of metabolites including gluconic acid (2.40-fold), 1,3,7-trimethyluric acid (9.78-fold), and melatonin (52.44-fold) collectively established a multi-layered antioxidant network (Lee et al., 2021). The accumulation of 2-isopropylmalic acid (2.89-fold), an intermediate in leucine biosynthesis, and allysine (69.02-fold), an oxidative derivative of lysine, further confirms enhanced protein hydrolysis and amino acid metabolism, providing a material basis for umami perception. Flavin adenine dinucleotide and alpha-ketoglutaric acid were reduced to 0.30- and 0.41-fold, respectively, with their consumption reflecting the demand for energy and amino acid synthesis required for rapid proliferation of LAB. These differential metabolites were mainly enriched in pathways such as the TCA cycle, glyoxylate and dicarboxylate metabolism, carbon fixation pathways in prokaryotes, arginine biosynthesis, and alanine, aspartate, and glutamate metabolism (Fig. 5-E).

Comparing group C with group B, a total of 18 differential metabolites were identified, including 8 upregulated and 10 downregulated metabolites. Among the upregulated metabolites, pyridoxine 5′-phosphate (PLP), 4-hydroxybenzaldehyde, 3-hydroxybenzaldehyde, and benzoic acid were increased by 3.58-, 5.72-, 4.49-, and 3.75-fold, respectively. PLP, as an essential coenzyme for glutamate decarboxylase, directly supports the higher GABA synthesis efficiency in group C (Yogeswara et al., 2020). Benzoic acid and its hydroxy derivatives exhibit antibacterial and antioxidant activities (Farhat et al., 2022). Their upregulation may reflect the metabolic transformation of polyphenolic compounds from peanut sprout juice by the compound microbial agent. High-molecular-weight polyphenols were converted into low-molecular-weight phenolic acids, thereby reducing the concentration of free polyphenols and alleviating astringency and bitterness. This finding is consistent with the electronic tongue results. Among the downregulated metabolites, taxa-4(20),11-dien-5-alpha-yl acetate, phosphorylcholine, glycerophosphoethanolamine, and phosphodimethylethanolamine, which are key intermediates in glycerophospholipid metabolism, were significantly decreased to 0.40-, 0.42-, 0.40-, and 0.44-fold, respectively. The downregulation of plant-derived secondary metabolites (e.g., taxa-4(20),11-dien-5-alpha-yl acetate) further confirms the metabolic transformation of flavor precursors from peanut sprout juice by the compound microbial agent. Meanwhile, the downregulation of phospholipid intermediates indicates that phospholipid metabolism was reprogrammed to meet the requirements of cell membrane composition under stress conditions (Baburina & Jackowski, 1999). These differential metabolites were mainly enriched in pathways including chlorocyclohexane and chlorobenzene degradation, glycerophospholipid metabolism, benzoate degradation, phenylalanine metabolism, and alanine, aspartate, and glutamate metabolism (Fig. 5-F).

**Fig. 6:**
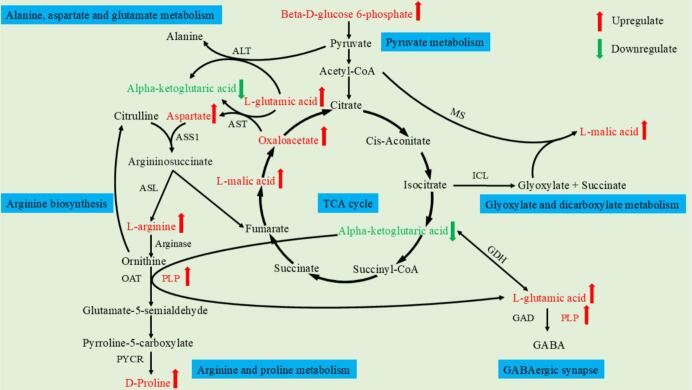
The key metabolic pathways of co-fermentation of selenium-enriched peanut sprouts and compound starter. ALT = Alanine aminotransferase, AST = Aspartate aminotransferase, ASS1 = Argininosuccinate Synthetase, ASL = Argininosuccinate lyase, PLP = Pyridoxal-5′-phosphate, OAT = Ornithine Aminotransferase, PYCR = Pyrroline-5-carboxylate Reductase, GDH = Glutamate dehydrogenase, GAD = Glutamate Decarboxylase, GABA = Gamma-aminobutyric acid, ICL = Isocitrate lyase, MS = Malate synthase.

Fig. 6 summarizes the key metabolic pathways and the interconnections among differential metabolites in Group C. In the TCA cycle, oxaloacetate and L-malic acid are significantly upregulated. Oxaloacetate is transaminated by aspartate aminotransferase (AST) to generate alpha-ketoglutaric acid and aspartate. Beta-d-glucose-6-phosphate enters glycolysis to produce pyruvate, which can be converted into acetyl-CoA to fuel the TCA cycle, or together with L-glutamic acid, be converted by alanine aminotransferase (ALT) into alpha-ketoglutaric acid and alanine. Aspartate and citrulline are condensed by argininosuccinate synthetase (ASS1) to form argininosuccinate, which is then cleaved by argininosuccinate lyase (ASL) into L-arginine and fumarate. L-arginine further promotes the synthesis of D-proline via the intermediates ornithine, glutamate-5-semialdehyde and pyrroline-5-carboxylate. In the glyoxylate shunt, isocitrate is cleaved by isocitrate lyase (ICL) to generate glyoxylate and succinate, while acetyl-CoA condenses with glyoxylate via malate synthase (MS) to produce L-malic acid, thereby enhancing the anaplerotic flux. Alpha-ketoglutaric acid is reversibly converted to L-glutamic acid by glutamate dehydrogenase (GDH). L-Glutamic acid, as a direct precursor of GABA, is then converted to GABA by glutamate decarboxylase (GAD) with PLP as an essential cofactor. In summary, Fig. 6 reveals the regulatory network rewired by the co-fermentation of peanut sprout juice and the compound microbial agent, involving the TCA cycle, the glyoxylate shunt, and amino acid metabolism. This network increases the accumulation of umami-related amino acids and promotes the synthesis of stress-protective metabolites, thereby explaining the metabolic basis for the improved flavor quality and enhanced bacterial adaptability of yogurt in Group C.

## 4 Conclusion

This study investigated the effect of a Se-enriched *Limosilactobacillus fermentum* CGMCC 17434 compound microbial agent synergising with peanut sprouts on the flavor of Se-enriched yogurt. The co-fermentation system achieved a high viable count of 8.70 lg CFU/mL, reduced pH, and increased TA, while improved WHC, texture and rheological properties, effectively compensating for the dilution effect of peanut sprout juice on gel strength. Flavor profiling revealed significant upregulation of fruity esters (pentanoic acid methyl ester and acetic acid ethyl ester) and aroma compounds (anethole, and 1-hexanol), concomitant with marked reductions in off-flavor compounds such as cyclopentanone. Metabolomic analysis further elucidated that these benefits arise from metabolic reprogramming in amino acid metabolism and the TCA cycle, coupled with phospholipid remodeling and antioxidant network activation, which collectively enhance bacterial adaptability and functionality. Future studies focusing on sensory evaluation and storage stability would be valuable to further assess the practical potential of this product. This is of great significance for the development of plant-based yogurt with high viable counts, selenium-enriched functionality, and unique flavor.

## CRediT authorship contribution statement

**Zhuhui Tian:** Writing – original draft, Visualization, Methodology, Data curation, Conceptualization. **Hui Zhou:** Visualization. **Xiefei Li:** Investigation. **Jie Luo:** Formal analysis. **Yuxing Guo:** Writing – review & editing. **Zhen Wu:** Methodology, Data curation. **Daodong Pan:** Methodology. **Xiankang Fan:** Writing – review & editing, Project administration, Funding acquisition, Conceptualization.

## Declaration of competing interest

The authors declare that they have no known competing financial interests or personal relationships that could have appeared to influence the work reported in this paper.

## Data Availability

Data will be made available on request.

## References

[bb0005] Atik D.S., Öztürk H.İ., Akın N. (2024). Perspectives on the yogurt rheology. International Journal of Biological Macromolecules.

[bb0010] Baburina I., Jackowski S. (1999). Cellular responses to excess phospholipid. Journal of Biological Chemistry.

[bb0015] Bai M., Yang S., Zhao Q., Wang D., Zhang T., Kwok L.Y., Sun Z. (2024). Fermentation characteristics of *Lactobacillus delbrueckii* subsp. *bulgaricus* T50 and *Streptococcus thermophilus* S10 complex starter: Enhancing fermentation performance, metabolic interaction, and storage stability. LWT.

[bb0020] Bai S., Zhang M., Tang S., Li M., Wu R., Wan S., Chen L., Wei X., Feng S. (2024). Effects and impact of selenium on human health, a review. Molecules.

[bb0025] Banerji R., Iyer P., Bhagwat A., Saroj S.D. (2022). Spermidine promotes lysozyme tolerance and acid stress resistance in streptococcus pyogenes M3. Microbiology.

[bb0030] Bao C., Yan M., Diao M., Anastasiia U., Zhang X., Zhang T. (2025). Effect of Ganoderma lucidum water extract on flavor volatiles and quality characteristics of set-type yogurt. Food Chemistry.

[bb0035] Benchabane G., Benchabane A., Benslimane A., Lamamra K., Yagoub M., Mahbubul I.M., Bekkour K. (2024). Rheological quality control tests of commercial strawberry stirred yogurt: pseudo-chewing process. Journal of Food Quality and Hazards Control..

[bb0040] Braga A.R.C., de Souza Mesquita L.M., Martins P.L.G., Habu S., de Rosso V.V. (2018). Lactobacillus fermentation of Jussara pulp leads to the enzymatic conversion of anthocyanins increasing antioxidant activity. Journal of Food Composition and Analysis.

[bb0045] Brüls M., Foroutanparsa S., Maljaars C.E.P., Olsthoorn M., Tas R.P., Voets I.K. (2024). Investigating the impact of exopolysaccharides on yogurt network mechanics and syneresis through quantitative microstructural analysis. Food Hydrocolloids.

[bb0050] Cai Z., Zhou S., Zhang T., Du Q., Tu M., Wu Z., Zeng X., Dang Y., Liu Z., Pan D., Liu Q. (2025). Synergistic enhancement of bio-yogurt properties by *Lactiplantibacillus plantarum* NUC08 and mulberry fruit extract. Food Chemistry.

[bb0055] Cheng H. (2010). Volatile flavor compounds in yogurt: A review. Critical Reviews in Food Science and Nutrition.

[bb0060] Cheng J.R., Liu X.M., Chen Z.Y., Zhang Y.S., Zhang Y.H. (2016). Mulberry anthocyanin biotransformation by intestinal probiotics. Food Chemistry.

[bb0065] Daszkiewicz T., Michalak M., Śmiecińska K. (2024). A comparison of the quality of plain yogurt and its analog made from coconut flesh extract. Journal of Dairy Science.

[bb0070] D’auria M., Lorenz R., Mecca M., Racioppi R., Romano V.A., Viggiani L. (2022). The scent of Neotinea orchids from Basilicata (southern Italy). Natural Product Research.

[bb0075] Dhakal R., Bajpai V.K., Baek K.H. (2012). Production of GABA (γ-aminobutyric acid) by microorganisms: A review. Brazilian Journal of Microbiology.

[bb0080] Dinh Q.T., Cui Z., Huang J., Tran T.A.T., Wang D., Yang W., Liang D. (2018). Selenium distribution in the Chinese environment and its relationship with human health: A review. Environment International.

[bb0085] El-Sayed H.S., El-Sayed S.M., Youssef A.M. (2022). Designated functional microcapsules loaded with green synthesis selenium nanorods and probiotics for enhancing stirred yogurt. Scientific Reports.

[bb0090] Fan X., Li X., Du L., Li J., Xu J., Shi Z., Pan D. (2022). The effect of natural plant-based homogenates as additives on the quality of yogurt: A review. Food Bioscience.

[bb0095] Fan X., Liu M., Shi Z., Zhang T., Du L., Wu Z., Zeng X., Wu X., Pan D. (2024). Binary probiotic fermentation promotes signal (cyclic AMP) exchange to increases the number of viable probiotics, anthocyanins and polyphenol content, and the odor scores of wolfberry fermented beverages. Food Chemistry.

[bb0100] Fan X., Zhang A., Zhang T., Tu M., Du Q., Ling N., Pan D. (2024). Effects of semen *Ziziphi Spinosae* extract and binary probiotics co-fermentation on the quality of yogurt and their underlying molecular mechanisms. Food Chemistry: X.

[bb0105] Farhat L.B., Aissaoui N., Torrijos R., Luz C., Meca G., Abidi F. (2022). Correlation between metabolites of lactic acid bacteria isolated from dairy traditional fermented Tunisian products and antifungal and antioxidant activities. Journal of Applied Microbiology.

[bb0110] Genc B., Karatas S.M., Tunç M.T. (2024). *Enterococcus durans* SL70, a novel exopolysaccharide producer from traditional sourdough fermentation of einkorn (Triticum monococcum L. ssp. monococcum). Food Technology and Biotechnology.

[bb0115] Godoi F.C., Ningtyas D.W., Geoffroy Z., Prakash S. (2021). Protein-based hydrocolloids: Effect on the particle size distribution, tribo-rheological behaviour and mouthfeel characteristics of low-fat chocolate flavored milk. Food Hydrocolloids.

[bb0120] Goldman R.C., Zakula D., Capobianco J.O., Sharpe B.A., Griffin J.H. (1996). Inhibition of 2, 3-oxidosqualene-lanosterol cyclase in Candida albicans by pyridinium ion-based inhibitors. Antimicrobial Agents and Chemotherapy.

[bb0125] Gu Y., Li X., Xiao R., Dudu O.E., Yang L., Ma Y. (2020). Impact of Lactobacillus paracasei IMC502 in coculture with traditional starters on volatile and non-volatile metabolite profiles in yogurt. Process Biochemistry.

[bb0130] Guan N., Liu L. (2020). Microbial response to acid stress: Mechanisms and applications. Applied Microbiology and Biotechnology.

[bb0135] Guo X., Cui H., Zhang H., Li Y., Cai J., Wei Q., Mao S., Sun M., Zhang X., Feng J., Li A., Dong J., Zhao Z., Wang J., Liu J., Hu Z. (2025). Effects of selenium supplementation on ornithine carbamoyl-transferase activity, the metabolisms of associated amino acids and fermentation flavors of *Levilactobacillus brevis*. Food Microbiology.

[bb0140] Gurkan H.A.C.E.R., Hayaloglu A.A. (2017). Volatiles and sensory characteristics of yogurt manufactured by incorporating basil (*Ocimum basilicum* L.). International Journal of Food Properties.

[bb0145] Han Y., Jin X., Zhang Z., Sun Q. (2025). Characterization of pectin with different structural features and its effects on yogurt quality. LWT.

[bb0150] Heiden-Hecht T., Wu B., Appavou M.S., Förster S., Frielinghaus H., Holderer O. (2023). Multiscale structural insight into dairy products and plant-based alternatives by scattering and imaging techniques. Foods.

[bb0155] Hou X., Lin L., Li K., Jiang F., Qiao D., Zhang B., Xie F. (2024). Towards superior biopolymer gels by enabling interpenetrating network structures: A review on types, applications, and gelation strategies. Advances in Colloid and Interface Science.

[bb0160] Koca N., Karadeniz F., Burdurlu H.S. (2007). Effect of pH on chlorophyll degradation and colour loss in blanched green peas. Food Chemistry.

[bb0165] Lai X., Li Y., Lan W., Zhao L., Wang K., Hu Z., Liu X. (2025). Interactions between proteins and polyphenols in plant-based food: Insight of allergenicity and off-flavor reduction. Food Chemistry.

[bb0170] Lee M., Song J.H., Choi E.J., Yun Y.R., Lee K.W., Chang J.Y. (2021). UPLC-QTOF-MS/MS and GC-MS characterization of phytochemicals in vegetable juice fermented using lactic acid bacteria from kimchi and their antioxidant potential. Antioxidants.

[bb0175] Li H., Tu M., Wu Z., Zeng X., Wu J., Pan D., Du Q. (2025). Comparison of gelation of legume protein and milk protein fermented by mixed starter cultures: Texture, rheological properties and protein structure. Food Chemistry: X.

[bb0180] Li R., He Z., Yan W., Yu H., Yi X., Sha Y., Zhang Q., Cai R., Pang W. (2023). Tricaprylin, a medium-chain triglyceride, aggravates high-fat diet-induced fat deposition but improves intestinal health. Food & Function.

[bb0185] Luo J., Tian Z., Yuan W., Peng X., Zhou H., Shen Q., Luo Y., Guo Y., Shi Z., Jiang X., Chen B., Pan D., Fan X. (2025). Anti-aging effect of *Limosilactobacillus fermentum* CGMCC 17434 in mice fed with fermented selenium-enriched yogurt. Food Bioscience.

[bb0190] Luo L., Zhang J., Zhang K., Wen Q., Ming K., Xiong H., Ning F. (2021). Peanut selenium distribution, concentration, speciation, and effects on proteins after exogenous selenium biofortification. Food Chemistry.

[bb0195] Luo W., Zhuang Y., Sun L., Gu Y., Ding Y., Fan X. (2025). Regulation of proline on *Lacticaseibacillus rhamnosus* cells under sodium lactate mediated osmotic stress: Resistance and underlying mechanisms. Food Research International.

[bb0200] Martínez F.G., Moreno-Martin G., Pescuma M., Madrid-Albarrán Y., Mozzi F. (2020). Biotransformation of selenium by lactic acid bacteria: Formation of seleno-nanoparticles and seleno-amino acids. Frontiers in Bioengineering and Biotechnology.

[bb0205] Molina G.E.S., Ras G., da Silva D.F., Duedahl-Olesen L., Hansen E.B., Bang-Berthelsen C.H. (2025). Metabolic insights of lactic acid bacteria in reducing off-flavors and antinutrients in plant-based fermented dairy alternatives. Comprehensive Reviews in Food Science and Food Safety.

[bb0210] Nguyen H.T., Afsar S., Day L. (2018). Differences in the microstructure and rheological properties of low-fat yoghurts from goat, sheep and cow milk. Food Research International.

[bb0215] Nicolotti C., Cirlini M., Del Vecchio L., Hadj Saadoun J., Bernini V., Gatti M., Martelli F. (2025). Lactic acid fermentation of *Chlorella vulgaris* to improve the aroma of new microalgae-based foods: Impact of composition and bacterial growth on the volatile fraction. Foods.

[bb0220] Oliveira I.C., de Paula Ferreira I.E., Casanova F., Cavallieri A.L.F., Lima Nascimento L.G., de Carvalho A.F., Nogueira Silva N.F. (2022). Colloidal and acid gelling properties of mixed milk and pea protein suspensions. Foods.

[bb0225] Peng C., Yao G., Sun Y., Guo S., Wang J., Mu X., Sun Z., Zhang H. (2022). Comparative effects of the single and binary probiotics of *Lacticaseibacillus casei* Zhang and *Bifidobacterium lactis* V9 on the growth and metabolomic profiles in yogurts. Food Research International.

[bb0230] Pophaly S.D., Singh P., Kumar H., Tomar S.K., Singh R. (2014). Selenium enrichment of lactic acid bacteria and *bifidobacteria*: A functional food perspective. Trends in Food Science & Technology.

[bb0235] Raj D.R.K., Gonçalves M.H., de Medeiros A.C., Bolini H.M.A., Riul A., Barbin D.F. (2025). Impedimetric multi-sensor system with gold and silver nanoparticles applied for basic taste assessment compared with human threshold method sensory analysis. Food Chemistry.

[bb0240] Ramos I.M., Seseña S., Poveda J.M., Palop M.L. (2023). Screening of lactic acid bacteria strains to improve the properties of non-fat set yogurt by in situ EPS production. Food and Bioprocess Technology.

[bb0245] Rohloff J., Bones A.M. (2005). Volatile profiling of Arabidopsis thaliana–putative olfactory compounds in plant communication. Phytochemistry.

[bb0250] Routray W., Mishra H.N. (2011). Scientific and technical aspects of yogurt aroma and taste: A review. Comprehensive Reviews in Food Science and Food Safety.

[bb0255] Ryan E., Galvin K., O’Connor T.P., Maguire A.R., O’Brien N.M. (2007). Phytosterol, squalene, tocopherol content and fatty acid profile of selected seeds, grains, and legumes. Plant Foods for Human Nutrition.

[bb0260] Shimamura T., Nishimura T., Iwasaki A., Odake S., Akuzawa R. (2009). Degradation of a bitter peptide derived from casein by lactic acid bacterial peptidase. Food Science and Technology Research.

[bb0265] Song I., Jeon H., Priatama R.A., Gayathri S., Ko K., Lee Y.K. (2023). Effect of plasma-activated water on peanut seed germination and vegetative growth in a hydroponic system. Plant Biotechnology Reports.

[bb0270] Spaccasassi A., Ye L., Rincón C., Börner R.A., Bogicevic B., Glabasnia A., Dawid C. (2024). Sensoproteomic characterization of *Lactobacillus Johnsonii*-fermented pea protein-based beverage: A promising strategy for enhancing umami and kokumi sensations while mitigating bitterness. Journal of Agricultural and Food Chemistry.

[bb0275] Teixeira J.S., Seeras A., Sanchez-Maldonado A.F., Zhang C., Su M.S.W., Gänzle M.G. (2014). Glutamine, glutamate, and arginine-based acid resistance in Lactobacillus reuteri. Food Microbiology.

[bb0280] Tian H., Shen Y., Yu H., He Y., Chen C. (2017). Effects of 4 probiotic strains in coculture with traditional starters on the flavor profile of yogurt. Journal of Food Science.

[bb0285] Tian H., Yu B., Yu H., Chen C. (2020). Evaluation of the synergistic olfactory effects of diacetyl, acetaldehyde, and acetoin in a yogurt matrix using odor threshold, aroma intensity, and electronic nose analyses. Journal of Dairy Science.

[bb0290] Utaida T., Moongngarm A., Itsaranuwat P. (2025). Co-cultivation of yoghurt bacteria with probiotics increased melatonin content and enhanced the antioxidant activity of soy milk yoghurt. Food Technology and Biotechnology.

[bb0295] Wang L., Liu Q., Li Y., Shi C., Zhang Y., Wang P., Zhang H., Wang R., Zhang W., Wen P. (2025). Revealing the impact of organic selenium-enriched *Lactiplantibacillus plantarum* NML21 on yogurt quality through volatile flavor compounds and untargeted metabolomics. Food Chemistry.

[bb0305] Wang M.S., Fan M., Zheng A.R., Wei C.K., Liu D.H., Thaku K., Wei Z.J. (2023). Characterization of a fermented dairy, sour cream: Lipolysis and the release profile of flavor compounds. Food Chemistry.

[bb0300] Wang L., Yu L., Zhang Y., Wu Z. (2026). Synergistic mechanisms of ultrasound and slightly acidic electrolyzed water in peanut germination revealed by multi-omics analysis. Ultrasonics Sonochemistry.

[bb0315] Wu B., Guo Y., Hao L., Zuo K., Du Y., An R., Wang B. (2025). The effects of *Leuconostoc mesenteroides* RSG7 exopolysaccharide on the physicochemical properties and flavor compounds of set yoghurt. Processes.

[bb0320] Wu T., Deng C., Luo S., Liu C., Hu X. (2023). Effect of rice bran on properties of yogurt: Comparison between addition of bran before fermentation and after fermentation. Food Hydrocolloids.

[bb0325] Wu Z., Chen T., Pan D., Zeng X., Guo Y., Zhao G. (2021). Resveratrol and organic selenium-rich fermented milk reduces D-galactose-induced cognitive dysfunction in mice. Food & Function.

[bb0330] Xi Y., Ikram S., Zhao T., Shao Y., Liu R., Song F., Ai N. (2023). 2-heptanone, 2-nonanone, and 2-undecanone confer oxidation off-flavor in cow milk storage. Journal of Dairy Science.

[bb0335] Xu X., Qiao W., Dong Y., Yang H., Xu H., Qiao M. (2025). 2, 3-butanediol dehydrogenase is more efficient than acetoin reductase at metabolizing reserve carbon to improve carbon cycling pathways in Lactococcus lactis N8. International Journal of Biological Macromolecules.

[bb0340] Yang H., Hao L., Yao S., Zhou R., Wu C. (2025). Rational engineering of arginine deiminase to enhance the acid tolerance of lactic acid bacteria. Journal of Agricultural and Food Chemistry.

[bb0345] Yang S., Zhao Q., Wang D., Zhang T., Zhong Z., Kwok L.Y., Sun Z. (2024). The interaction between *Lactobacillus delbrueckii* ssp. *bulgaricus* M-58 and *Streptococcus thermophilus* S10 can enhance the texture and flavor profile of fermented milk: Insights from metabolomics analysis. Journal of Dairy Science.

[bb0350] Yao Y., Zheng Y., Dai H., Jia Y., Li C. (2024). Kinetics of squalene quenching singlet oxygen and the thermal degradation products identification. Journal of Agricultural and Food Chemistry.

[bb0355] Yogeswara I.B.A., Maneerat S., Haltrich D. (2020). Glutamate decarboxylase from lactic acid bacteria—A key enzyme in GABA synthesis. Microorganisms.

[bb0360] Yohannes E., Thurber A.E., Wilks J.C., Tate D.P., Slonczewski J.L. (2005). Polyamine stress at high pH in Escherichia coli K-12. BMC Microbiology.

[bb0365] Yu M., Ma J., Wang X., Lu M., Fu X., Zhang L., Shi T., Xu L., Zhang L., Xie T. (2022). Peanut sprout yogurt: Increased antioxidant activity and nutritional content and sensory evaluation by fuzzy mathematics. Journal of Food Processing and Preservation.

[bb0370] Yuan L., Yuan J., Gao C., Zhao H., Wu C., Yang Z.H. (2025). *Lactiplantibacillus plantarum S1 as a novel dual-functional probiotic strain for high-efficiency organoselenium biotransformation in functional food development*. Foods.

[bb0375] Yuan W., Zhou H., Chen J., Wu S., Yan F., Li M., Fan X. (2026). Characterization of the effect of *Levilactobacillus brevis* CGMCC 1.5954 combined with *Lactiplantibacillus plantarum* subsp. *plantarum* CGMCC 1.5953 on the red bean sprouts GABA-enriched fermented milks. Food Chemistry.

[bb0380] Zan L., Chen Z., Zhang B., Zou X., Lan A., Zhang W., Yuan Y., Yue T. (2024). Screening, characterization and probiotic properties of selenium-enriched lactic acid bacteria. Fermentation.

[bb0385] Zhang W., Zhang Y., Wang S., Wei K., Liu S., Liu M., Yu H. (2025). Abiotic stress-induced resveratrol accumulation in peanut sprouts: Methods and research frontiers. Plant Cell, Tissue and Organ Culture (PCTOC).

[bb0390] Zhou R., Li Z., Qi X., Li L., Zhang X., Diao M., Zhang T. (2025). Effects of protein fortifiers on physicochemical and sensory properties of set yogurt supplemented with ginseng extract. Journal of Dairy Science.

[bb0395] Zhou Z., Fan Z., Meenu M., Xu B. (2021). Impact of germination time on resveratrol, phenolic acids, and antioxidant capacities of different varieties of peanut (Arachis hypogaea Linn.) from China. Antioxidants.

[bb0400] Zhu T., Pan Q., Xiao K., Zuo C., Liu Q., Zhou D., Tu K. (2024). Stilbenes-enriched peanut sprouts alleviated physical fatigue via regulating interactions of nutrients–microbiota–metabolites revealed by multi-omics analysis. Food & Function.

[bb0405] Zou B., Zheng X., Sun R., Na X., Du M., Wu C. (2025). Accelerated gelation and strengthened network formation in whey-pea binary protein systems via linear or branched β-glucans. Carbohydrate Polymers.

