# Supplementary material for: Effect of a Se-enriched Limosilactobacillus fermentum CGMCC 17434 compound microbial agent synergising with peanut sprouts on the flavor of Se-enriched yogurt
Source: Food Chem X. 2026 Jun 23;37:104132. doi: 10.1016/j.fochx.2026.104132 (PMC13320279; doi:10.1016/j.fochx.2026.104132)
Supplement: Supplementary file 1 — Supplementary material [file mmc1.docx]

Supplementary Table S1

| Compound | Odor description | Threshold (μg/L) | OAV | | |
| --- | --- | --- | --- | --- | --- |
|  |  |  | A | B | C |
| Acetoin | Butter, creamy, green pepper | 55 | <1 | <1 | <1 |
| 1-Hexanol | Green, fruity, apple‑skin, oily | 5.6 | <1 | <1 | 13.77 ± 7.41 |
| 2-Heptanone | Cheese, fruity, coconut | 5 | - | <1 | <1 |
| 2-Nonanone | Cheesy, green, fruity | 5 | 6.06 ± 2.94 | 6.03 ± 1.26 | 5.07 ± 0.57 |
| 2-Undecanone | Fresh, green, orange, rose | 5.5 | 4.11 ± 2.01 | <1 | - |
| Nonanal | Aldehydic citrus, cucumber, watermelon rind | 1 | 47.64 ± 10.41 | 19.47 ± 1.95 | 59.55 ± 2.19 |
| Decanal | Floral, orange peel | 3 | <1 | <1 | 25.71 ± 13.83 |
| Dodecanal | Soapy, waxy, citrus | 1.5 | - | 7.83 ± 0.09 | 3.93 ± 0.39 |
| Octanoic acid | Cheesy, sweaty | 500 | <1 | - | <1 |
| Hexanoic acid | Cheese, rancid, floral | 35.6 | <1 | - | - |

Odor activity values (OAVs) of volatile compounds in yogurt. Values are expressed as mean ± standard deviation (n = 3). “-” indicates that the compound was not detected. OAV <1 indicates that the compound is unlikely to contribute significantly to the overall aroma.
